# EPA 2024, Budapest - Abstract - ePoster Viewing Digital Psychiatric Innovations from a Business Perspective – new era, new business models

**DOI:** 10.1192/j.eurpsy.2024.1704

**Published:** 2024-08-27

**Authors:** B. Kiss, T. Kurimay, B. Nagy

**Affiliations:** ^1^ Semmelweis University; ^2^Syreon Research Institute, Budapest, Hungary

## Abstract

**Introduction:**

The advent of digital innovations in psychiatry has ushered in a new era in mental healthcare. These innovations offer the potential for enhanced diagnosis, treatment, and patient care. Establishing scientifically backed, dynamic, and adaptive business models is necessary to launch sustainable innovations onto the healthcare market.

**Objectives:**

This poster aims to provide a comprehensive understanding of the complex business-related challenges posed by digital innovations in psychiatry and to offer insights into potential strategies to address these challenges. The objectives include illuminating the dynamic landscape of digital psychiatric care from a business perspective.

**Methods:**

A systematic review of the current literature was conducted, encompassing scholarly articles, industry reports, and expert perspectives. This method enabled the synthesis of insights regarding how digital innovations are reshaping the business models of psychiatric medical markets and the unique challenges.

**Results:**

Digital innovations in psychiatry are catalyzing a transformation of business models in the field. Telepsychiatry, Digital platforms, VR technologies, and AI-driven diagnostic tools have expanded the reach of psychiatric services, potentially attracting new patient populations and offering innovative payment models. The opportunities presented by these technologies are promising. However, substantial challenges exist in parallel. Safeguarding data privacy and security is paramount, given the sensitive nature of patient information. Navigating the evolving regulatory, managing the costs associated with the adoption and maintenance of these technologies pose significant hurdles. Complex pricing structures and reimbursement models further add to the complexity of the challenges, necessitating adaptability and innovative strategies.

**Conclusions:**

This poster underscores the dynamic and multifaceted nature of business models in the market of psychiatric innovations. While these innovations offer expanded service reach, improved patient engagement, potential for innovative payment models, addressing the business-related challenges is of utmost importance. Compliance with data privacy regulations, cost management, adaptability in pricing and reimbursement strategies are fundamental for psychiatric innovators. Proactive measures are pivotal as the mental healthcare field continues to embrace digital innovations. By addressing these challenges, the mental health industry can fully harness the transformative potential of these innovations to enhance patient care, improve access to services, and ensure the sustainability of high-quality psychiatric care. The evolving business models in psychiatry require astute management and innovation to thrive in this digital era.

**Disclosure of Interest:**

None Declared

